# Infantile Spasms (West Syndrome): Integrating Genetic, Neurotrophic, and Hormonal Mechanisms Toward Precision Therapy

**DOI:** 10.3390/medicina61122223

**Published:** 2025-12-16

**Authors:** Bibigul Abdygalyk, Marat Rabandiyarov, Marzhan Lepessova, Gaukhar Koshkimbayeva, Nazira Zharkinbekova, Latina Tekebayeva, Azamat Zhailganov, Alma Issabekova, Bakhytkul Myrzaliyeva, Assel Tulendiyeva, Assem Kurmantay, Arailym Turmanbetova, Sandugash Yerkenova

**Affiliations:** 1 Department of Neurology, Kazakhstan’s Medical University “KSPH”, Almaty 050060, Kazakhstan; kalkaman061182@gmail.com; 2 Children’s City Clinical Hospital No. 2, Almaty 050060, Kazakhstan; dadi_2004@mail.ru (M.R.); azamatzhailganov@gmail.com (A.Z.); assel_tulendieva@mail.ru (A.T.); 3 Department of General Medical Practice with Courses, Kazakh-Russian Medical University, Almaty 050060, Kazakhstan; mar.lepessova@gmail.com (M.L.); alma_64@mail.ru (A.I.); myrzaliyeva@gmail.com (B.M.); asem_448_@mail.ru (A.K.); arailym.lmn@gmail.com (A.T.); 4 Department of Neurology, South Kazakhstan Medical Academy, Shymkent 160000, Kazakhstan; nazirazhar@mail.ru; 5 “University Medical Center” Corporate Fund, Turan Street 32, Astana 010000, Kazakhstan; lati-teckebaeva@yandex.kz

**Keywords:** infantile spasms, West syndrome, tuberous sclerosis complex, pathogenesis, molecular therapy

## Abstract

*Background and Objectives*: Infantile spasms (ISs), or West syndrome (WS), represent an early-onset epileptic encephalopathy in which diverse structural, genetic, metabolic, infectious, and neurocutaneous conditions converge on a shared pattern of hypsarrhythmia, clustered spasms, and later developmental impairment. Growing use of genomic diagnostics has revealed that variants in *STXBP1*, *KCNQ2*, *GRIN2A*, *GRIN2B*, and *TSC*-related genes are more common than previously recognized and can be linked to partially actionable pathways. This review aimed to synthesize current evidence on the multifactorial etiology, network-based pathogenesis, and evolving targeted therapies for ISs, with particular attention to *TSC*-related forms. *Materials and Methods*: A structured narrative review was undertaken of publications from 1990 to 2025 in PubMed, Scopus, Web of Science, and Embase using terms related to ISs, WS, genetics, mTOR, ACTH, vigabatrin, ketogenic diet, and precision therapies. Authoritative guidance from ILAE and AAN was incorporated. Clinical, molecular, and therapeutic data were grouped under etiological, pathogenetic, and management domains. *Results*: Structural causes remained the largest group, but combined genetic, genetic–structural, and metabolic etiologies accounted for about one third of contemporary cohorts. Early network disruption involving cortex, thalamus, basal ganglia, and brainstem, together with imbalances in NGF, BDNF, and IGF-1, explained why distinct primary insults produce a uniform electroclinical phenotype. Early treatment with ACTH or high dose prednisolone, with or without vigabatrin, was consistently associated with higher electroclinical remission and better developmental outcome. Everolimus and related mTOR inhibitors showed benefit in TSC-associated ISs, while agents directed at NMDA receptors or KCNQ channels are emerging for genotype defined subgroups. *Conclusions*: ISs should be approached as a heterogeneous but mechanistically convergent disorder in which rapid diagnosis, parallel genetic testing, and early disease modifying therapy improve prognosis. Integration of molecular profiling with standardized outcome monitoring is likely to move management from symptomatic seizure control to pathway-specific intervention.

## 1. Introduction

Infantile spasms (ISs), historically referred to as West syndrome (WS) following Dr. William James West’s delineation in 1841, constitute one of the most severe variants of early-onset epileptic encephalopathy [1,2,3]. This condition is characterized by a triad consisting of clustered epileptic spasms, hypsarrhythmia evident on electroencephalography (EEG), and either developmental stagnation or regression [4,5]. Despite the apparent uniformity in clinical presentations, the etiological underpinnings are surprisingly diverse, encompassing structural cerebral anomalies, chromosomal and single-gene mutations, metabolic disorders, immune system dysfunctions, and postnatal injuries [6,7,8,9]. The commonality among these varied origins is the premature disruption of cortical-subcortical circuitry during a critical neurodevelopmental phase, which results in abnormal network synchronization and the onset of epilepsy [10]. A significant aim of contemporary neurogenetic and translational research has been to elucidate the convergent biological mechanisms that yield this shared electroclinical phenotype [11].

Over the past twenty years, significant advancements have been made in mapping the genetic and molecular framework of ISs [12]. Cutting-edge sequencing methodologies, such as next-generation sequencing (NGS), whole-exome sequencing (WES), and genome-wide copy-number analysis, have identified pathogenic variants across more than one hundred genes, including *STXBP1*, *KCNQ2*, *GRIN2A*, *GRIN2B*, *ARX*, and *CDKL5* [13,14,15,16,17,18]. Numerous genes implicated in this condition encode proteins vital for neuronal migration, synaptic vesicle cycling, ion-channel modulation, and intracellular signaling pathways, thereby directly correlating molecular dysregulation with network instability [19]. Concurrently, investigations into *TSC1* and *TSC2* mutations have underscored the pathogenic significance of mTOR pathway hyperactivation in cortical dysplasia and epileptogenesis, thereby creating a mechanistic link between genetic and acquired etiologies [20,21,22]. These findings have transformed the understanding of WS from a strictly clinical entity to a continuum of molecularly characterized developmental and epileptic encephalopathies (DEEs) [23].

The prevalence of WS is approximated to be around 2–3.5 per 10,000 live births, representing nearly 10% of all epilepsy cases that manifest within the initial year of life [24]. Epidemiological investigations conducted in North America, Europe, and Asia consistently identify a peak incidence occurring between 3 and 7 months of age, with a slight male bias observed [25,26,27]. Structural and genetic factors collectively account for over 70% of instances, while idiopathic or cryptogenic variants comprise a diminishing minority as advancements in genetic diagnostics progress [28]. The prognosis is notably heterogeneous and largely influenced by underlying causes and the timing of therapeutic intervention. In spite of improvements in early identification, merely one-third of individuals attain long-term seizure remission, with approximately 50% encountering subsequent epileptic syndromes, such as Lennox–Gastaut syndrome or other developmental epileptic encephalopathies [29,30,31]. In areas with limited resources, delays in diagnosis and restricted availability of hormonal therapies further complicate developmental outcomes, highlighting the pressing necessity for standardized global treatment protocols.

At the pathophysiological level, ISs are increasingly conceptualized as a network disorder in which structural, metabolic, or genetic disturbances converge upon a restricted array of molecular pathways that regulate the excitatory–inhibitory equilibrium, neurotrophin expression, and neuroendocrine signaling [32,33]. Aberrations in GABAergic maturation, dysregulation of neurotrophic factors such as nerve growth factor (NGF) and insulin-like growth factor 1 (IGF-1), and mTOR-mediated hyperactivation represent recurring mechanistic patterns across various etiologies [34]. These observations clarify why therapeutic agents that act on different molecular pathways, including adrenocorticotropic hormone (ACTH), corticosteroids, vigabatrin, and the ketogenic diet, can each mitigate spasms by partially restoring the disrupted homeostatic balance. Simultaneously, neuroinflammatory and immunomodulatory mechanisms have garnered recognition as contributors to the process of epileptogenesis, thereby providing a basis for the effectiveness of corticosteroids and ACTH that extends beyond their endocrine actions [35,36,37].

In the last decade, therapeutic advancements have started to translate these mechanistic understandings into clinical practice [38]. Everolimus, an mTOR inhibitor, has shown clinical efficacy in WS associated with *TSC1*/*TSC2* mutations, while NMDA receptor antagonists, such as memantine, and potassium-channel openers, like retigabine, are currently being investigated for their potential in *GRIN2A*/*GRIN2B* and *KCNQ2*-related epileptic encephalopathies, respectively [39]. Adjunctive neurosteroid analogs (e.g., ganaxolone) and peptides derived from IGF-1 represent additional strategies aimed at modifying disease progression that may help restore neurochemical equilibrium and synaptic integrity [40]. These targeted approaches herald a transformative shift from merely symptomatic seizure management toward precision-guided therapies specifically tailored to the underlying molecular anomalies [41,42].

Notwithstanding significant progress, the clinical management of WS continues to present considerable challenges, with numerous infants still facing delays in diagnosis, inadequate seizure control, and severe developmental consequences. Furthermore, the expanding genetic landscape has surpassed the pace of clinical application, leading to ambiguity regarding which genetic findings should influence treatment decisions. Consequently, this review aims to integrate current insights into the multifactorial origins, molecular mechanisms, and novel therapeutic approaches associated with ISs syndrome. By amalgamating clinical, genetic, and translational research data, the objective is to develop a cohesive framework that correlates early neurological disruptions with distinct molecular pathways and to identify practical targets that may guide precision medicine. The ultimate aim is to connect mechanistic insights with clinical relevance, thereby enhancing early diagnostic capabilities, optimizing treatment strategies, and improving neurodevelopmental outcomes for affected children.

## 2. Materials and Methods

This comprehensive narrative review was conducted through a structured literature search and synthesis process integrating clinical, molecular, and therapeutic aspects of ISs and WS. Peer-reviewed publications in English were retrieved from PubMed, Scopus, Web of Science, and Embase databases between 1990 and 2025 using the following search terms and their Boolean combinations: “infantile spasms,” “West syndrome,” “infantile epileptic spasms syndrome,” “pathogenesis,” “genetic etiology,” “mTOR,” “ACTH,” “vigabatrin,” “ketogenic diet,” “IGF-1,” “neurosteroids,” “*TSC1*,” “*TSC2*,” “*STXBP1*,” “*KCNQ2*,” “*GRIN2A*,” “*GRIN2B*,” “neurotrophins,” “molecular therapy,” and “CRISPR.” Additional sources were identified through manual cross-referencing of citations from relevant reviews and clinical guidelines issued by the International League Against Epilepsy (ILAE) and American Academy of Neurology (AAN). Inclusion criteria encompassed original clinical studies, cohort analyses, case reports, translational and experimental investigations, and authoritative review articles addressing epidemiology, genetics, pathophysiology, diagnostic methods, and therapeutic outcomes in ISs. Articles focusing solely on unrelated epileptic encephalopathies or adult-onset epilepsies were excluded. Data were extracted, categorized, and synthesized under predefined thematic domains including etiologic classification, molecular mechanisms, neuroendocrine pathways, and therapeutic innovations. Genetic information was cross-validated using OMIM, ClinVar, and GeneCards databases, and gene symbols were formatted according to the HUGO Gene Nomenclature Committee (HGNC) standards. The manuscript integrates 210 cited references, ensuring comprehensive coverage of the historical, mechanistic, and emerging perspectives on ISs and facilitating a coherent framework for future translational and clinical research.

## 3. Multifactorial Etiology

**Figure 1:**
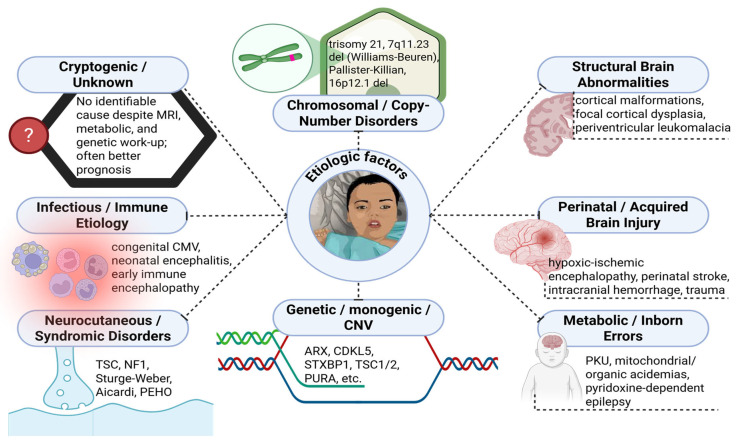
Major etiologic categories of WS [47,48,49,50,51,52,53]. This schematic summarizes the principal causes of infantile epileptic spasms syndrome, including structural brain abnormalities, genetic and monogenic disorders, chromosomal copy-number variations, metabolic/inborn errors, infectious or immune etiologies, neurocutaneous syndromes, perinatal/acquired brain injury, and cryptogenic cases with no identifiable cause despite thorough evaluation. Each category is illustrated with representative examples such as trisomy 21, TSC1/TSC2-related neurocutaneous disorders, and congenital infections. The diagram highlights the multifactorial nature of ISs and the convergence of diverse etiologies on a shared epileptic phenotype.

ISs, frequently referred to as WS, embody a cohesive clinical phenotype that may emerge from a diverse array of etiological mechanisms, which include but are not limited to structural, metabolic, infectious, immunological, and genetic anomalies (Figure 1). Similarly to other neurodevelopmental disorders characterized by seizures and developmental impairment, ISs may arise from a single pathogenic insult or from complex interactions among multiple contributing factors [43]. In certain instances, the etiological basis remains elusive despite exhaustive neuroimaging, metabolic, and genetic assessments, resulting in categorization as cryptogenic or of unknown origin, which is typically correlated with a relatively favorable prognostic outlook [44,45]. The ILAE has further delineated these classifications by differentiating between symptomatic, cryptogenic, and idiopathic subgroups of ISs, with the latter defined by a history of normal development prior to manifestation, unremarkable neuroimaging findings, and a hypsarrhythmic EEG pattern devoid of focal epileptiform discharges [46].

Symptomatic ISs are characterized by the presence of an identifiable etiology or an existing developmental delay at the moment of spasm onset. Approximately 60–70% of individuals are classified within this category [47,48]. The etiological spectrum of symptomatic ISs can be systematically categorized based on the timing of the inciting event into prenatal, perinatal, or postnatal origins, each of which corresponds to distinct pathogenic mechanisms. Prenatal etiologies encompass congenital anomalies of the central nervous system (CNS), including cortical dysplasia, lissencephaly, holoprosencephaly, and hemimegalencephaly, which collectively account for approximately 30% of all occurrences [49]. Neurocutaneous syndromes and other syndromic disorders frequently contribute to prenatal etiologies. Notably, tuberous sclerosis complex (TSC) represents the most prevalent condition, with around 68% of individuals diagnosed with TSC experiencing ISs [50,51]. Additionally, chromosomal and copy-number variations, such as trisomy 21, deletions on chromosome 7q11.23 associated with Williams–Beuren syndrome (WBS), and deletions on chromosome 16p12.1, are also substantial contributors, comprising up to 15% of prenatal etiological factors [52,53].

Recent advancements in molecular genetics have clarified that mutations in a variety of genes are directly associated with the pathogenesis of ISs. These encompass genes that encode syntaxin-binding protein 1 (*STXBP1*), calcium/calmodulin-dependent serine protein kinase (*CASK*), *ALG13*, and adenylosuccinate lyase [28,54,55]. Inborn metabolic errors constitute another significant etiological category, with a minimum of 25 metabolic disorders currently acknowledged. Among these, phenylketonuria persists as the most prevalent, particularly in areas where neonatal screening initiatives are not comprehensively enacted [56]. Furthermore, prenatal infectious agents, such as congenital toxoplasmosis, syphilis, cytomegalovirus infection, and Zika virus infection, also play a role in the complex etiological landscape of ISs [57].

Perinatal and postnatal occurrences represent additional pivotal factors. Hypoxic–ischemic encephalopathy and neonatal hypoglycemia rank among the most prevalent perinatal etiologies [58,59]. Moreover, low birth weight has been identified as a significant risk factor, manifesting three to four times more frequently in infants experiencing ISs in comparison to the general populace [60]. In the postnatal phase, traumatic injuries, perinatal strokes, central nervous system infections, and neoplasms have been associated, collectively accounting for 15–67% of symptomatic instances [61,62]. Importantly, numerous structural and acquired injuries exhibit convergent molecular and cellular mechanisms that overlap with genetic and metabolic disorders. For instance, the signaling pathways disrupted in tuberous sclerosis intersect with those implicated in cortical malformation syndromes and chromosomal anomalies of the brain, thereby exemplifying a shared pathogenic continuum [63].

Recent multicenter investigations have emphasized the increasing acknowledgment of genetic and molecular determinants as primary drivers or modifiers of ISs. In a cohort derived from the National Infantile Spasms Consortium, the etiological distribution among 161 patients with confirmed diagnoses indicated genetic etiologies in 14.4%, genetic–structural in 10.0%, structural–congenital in 10.8%, structural–acquired in 22.4%, metabolic in 4.8%, and infectious in 2% [64]. These observations underscore the manner in which contemporary diagnostic methodologies, including array-comparative genomic hybridization (array-CGH), NGS, WES, and whole-genome sequencing (WGS), are transforming our understanding of the pathogenesis of ISs [65,66,67]. The resultant molecular revelations propose that vascular, metabolic, and immune insults frequently operate against a genetically predisposed backdrop, thereby fortifying the notion of a multifactorial etiology that integrates both intrinsic and environmental influences [67,68].

### 3.1. Genetic and Molecular Determinants

Genetic causes represent one of the most extensively investigated etiologic groups in infantile epileptic spasms syndrome (IESS) (Table 1). As reported by Poeta et al. [69], the majority of genes implicated in IESS display considerable phenotypic heterogeneity, mirroring that observed in other DEEs. Familial clustering and concordance in monozygotic twins suggest that a genetic predisposition underlies many cases of IESS [69]. The earliest identified X-linked genes, *ARX* [69] and *CDKL5* [70], are both located on the short arm of the X chromosome and are highly expressed during fetal neurogenesis. Mutations in these genes disrupt neuronal migration and interneuron differentiation, leading to severe early-onset epileptic spasms with profound developmental delay. The discovery of *ARX* and *CDKL5* established the first molecular link between neurodevelopmental regulation and the IESS phenotype, highlighting the relevance of GABAergic circuit disruption in disease pathogenesis.

Subsequent studies expanded the genetic landscape to include several autosomal genes associated with cortical malformations [71,72,73,74,75,76,77]. Variants in *PAFAH1B1*/*LIS1* [71], *DCX* [72], and *TUBA1A* [73] were recognized as key contributors to neuronal migration disorders, such as lissencephaly and subcortical band heterotopia, which frequently manifest with spasms in infancy. The role of *STXBP1* [74] has been particularly emphasized as one of the most recurrent single-gene causes of IESS, associated with both classic hypsarrhythmia and broader epileptic encephalopathy spectra. Ion channel dysfunction also contributes significantly. Mutations in *KCNQ2* [75] cause neonatal epileptic encephalopathy with subsequent progression to IESS, while alterations in *GRIN2A* [77] and *GRIN2B* [78] affect NMDA-receptor signaling and glutamatergic transmission, underscoring the importance of excitatory–inhibitory balance in seizure generation. Similarly, *MAGI2* [76] deletions in the 7q11.23 region link synaptic scaffolding deficits with the onset of spasms.

Several other genes further illustrate the mechanistic diversity of IESS. Mutations in *FOXG1* [79], *NSD1* [80], and *SPTAN1* [81] affect transcriptional regulation, chromatin remodeling, and cytoskeletal integrity, respectively, contributing to microcephaly, hypomyelination, and dysmorphic features observed in affected infants. Copy number variations (CNVs) involving *NEDD4* [82] and *CALN1* [83] have been reported as potential risk factors, possibly influencing synaptic plasticity and neuronal excitability. Other genes such as *WDR45* [84], *RARS2* [85], *UBA5* [86], and *IARS2* [87] illustrate the contribution of mitochondrial and metabolic dysfunctions, in which impaired energy metabolism or defective protein turnover lowers seizure thresholds. These findings confirm that both structural and biochemical alterations converge on a shared epileptogenic phenotype, consistent with the multifactorial nature of IESS.

Advances in NGS have led to the identification of numerous novel and candidate genes associated with IESS. Studies employing WES and array-CGH have revealed mutations in *PHACTR1* [88], *ATP2A2* [89], *CD99L2* [90], *CLCN6* [91], *CYFIP1* [92], *CYFIP2* [93], *GNB1* [94], *GPT2* [95], and *HUWE1* [96], each implicated in synaptic regulation, ion transport, or intracellular trafficking. Furthermore, chromatin-modifying and cytoskeletal genes such as *KMT2D* [97] and *MYO18A* [98] have emerged as new contributors, particularly in syndromic forms with craniofacial or skeletal anomalies. The discovery of *NOS3* [99], *RYR1* [100], *RYR2* [101], and *RYR3* [102] variants underscores the growing recognition of calcium-signaling pathways in epileptogenesis. Mutations in *TAF1* [103] and *TECTA* [104] have been described in WES-based cohort studies, supporting transcriptional and extracellular matrix dysregulation as additional molecular mechanisms.

Recent studies have also highlighted de novo mutations in *PURA* [106], which cause PURA-associated neurodevelopmental disorder characterized by severe intellectual disability, hypotonia, and early-onset spasms. Voltage-gated sodium channel genes *SCN2A* [106], *SCN1A* [107], and *SCN8A* [108] have been increasingly recognized as major contributors to IESS, particularly in early-infantile epileptic encephalopathies. These mutations disrupt neuronal excitability and contribute to overlapping phenotypes that include Dravet-like and Lennox–Gastaut-like presentations. Autosomal recessive defects in *WWOX* [109] and mosaic or germline variants in *SLC35A2* [110] have been described in both structural and non-structural forms, the latter being associated with focal cortical dysplasia. Moreover, *NF1* [111] and *TSC2/TSC1* [112] remain among the most frequent syndromic causes of IESS, particularly when cortical tubers or subependymal nodules are evident on neuroimaging.

Finally, the expanding use of genome-wide sequencing continues to refine the genetic architecture of IESS. Emerging evidence points to *TOP2B* [113] as a novel candidate gene, with loss-of-function variants leading to neuronal maturation defects and cortical disorganization. Collectively, the growing list of genes listed in Table 1 highlights the remarkable etiologic diversity of WS, encompassing transcriptional regulators, ion channels, synaptic organizers, cytoskeletal proteins, and metabolic enzymes. This molecular heterogeneity reflects the convergence of distinct cellular pathways on a shared epileptic phenotype characterized by spasms and developmental arrest. As sequencing technologies and functional genomics evolve, it is expected that additional genes and network interactions will be uncovered, paving the way for improved diagnostic precision and genotype-guided therapeutic strategies.

### 3.2. Other Factors

Structural abnormalities of the developing brain represent a consistent and well-documented substrate for ISs. Malformations such as lissencephaly, focal cortical dysplasia, polymicrogyria, hydranencephaly [114], hemimegalencephaly, and other disorders of neuronal migration frequently precede the onset of spasms, since they disturb cortical organization and the balance of excitatory and inhibitory circuits. Classical lissencephaly is most often related to pathogenic variants in *PAFAH1B1/LIS1* and *DCX*, and in affected cohorts epileptic spasms have been reported in up to 80% of children with these genetic forms, which confirms that disruption of the microtubule and migration machinery has a strong epileptogenic effect in early life. Additional structural–genetic observations support this view. A de novo heterozygous mutation of *KIF2A* was identified in a child with lissencephaly, developmental delay, and ISs [115], showing that kinesin-related defects can converge on the same phenotype. Another infant with periventricular nodular heterotopia and ISs carried an unbalanced translocation involving 3p26.2–10p15.1 together with a 6q22.31 duplication, which indicates that combined chromosomal dosage changes may also produce a cortical dysgenesis–IS profile [116]. A further report described a child with a novel homozygous nonsense mutation in *B3GALNT2* who presented with Walker–Warburg syndrome, ISs, and sensorineural hearing loss, thereby linking defective glycosylation of α-dystroglycan to early epileptic spasms [117]. Taken together these observations show that very different developmental pathways, including cytoskeletal transport, vascular patterning, and O-mannosyl glycosylation, can all culminate in an ISs phenotype when cortical formation is severely disturbed.

ISs are also encountered within complex malformation or chromosomal syndromes. Down syndrome, Pallister–Killian syndrome, and WBS are the best characterized examples. In a series of 183 individuals with Down syndrome, 15 had epilepsy and 4 of them had ISs, which indicates that spasms occur in a non-negligible subset of these patients [118]. Tapp et al. [119] reported that seizures in Down syndrome occur in 1–13% of cases, and among these 6–32% present with ISs, suggesting that trisomy 21 creates a brain microenvironment that is permissive for early epileptogenesis. Pallister–Killian syndrome, which is caused by mosaic tetrasomy 12p, is characterized by dysmorphic facial features, pigmentary changes, alopecia, intellectual disability, and epilepsy; in these patients epileptic spasms tend to appear later than in classic WS [120]. In WBS, which is due to a 7q11.23 microdeletion, Marshall et al. [59] described a patient with ISs who had a larger deletion extending to 7q21.11 that included *MAGI2*, a synaptic scaffolding gene previously linked to ISs, indicating that enlargement of the deleted region may increase epileptic risk. Sporadic cases of ISs have also been described in Schinzel–Giedion, Smith–Lemli–Opitz, Smith–Magenis, Sotos, and Noonan-like syndromes with *PPP1CB* variants, showing that many multisystem developmental disorders can express spasms as an early neurological manifestation [121,122]. Two rare neurodevelopmental disorders, PEHO syndrome and Aicardi syndrome, often start clinically with spasms. PEHO is characterized by progressive encephalopathy, hypsarrhythmia, optic atrophy, and cerebellar atrophy resulting from granule cell loss; a mutation in *ZNHIT3* was recently identified as the main genetic cause [123]. Aicardi syndrome affects only females and is defined by agenesis of the corpus callosum, chorioretinal lacunae, severe intellectual disability, ISs, and frequently asymmetric hypsarrhythmia; intracranial tumors of the choroid plexus have also been reported [124]. These syndromic associations underscore that ISs may be the earliest recognizable sign of a complex disorder and that targeted genetic testing is indicated when dysmorphic or multiorgan features accompany spasms.

Inborn errors of metabolism constitute another relevant etiologic group and should always be considered in infants with spasms, especially when neuroregression, movement disorders, or multiorgan involvement are present. Classical phenylketonuria (PKU), caused by biallelic variants in the *PAH* gene and characterized biochemically by defective conversion of phenylalanine to tyrosine, was historically a common cause of ISs in the pre-screening era, with affected infants exhibiting hypopigmentation, progressive cognitive impairment, and seizures including spasms due to severe white matter and gray matter injury [125]. The rarer tetrahydrobiopterin deficiency forms, which affect the cofactor of phenylalanine hydroxylase, show an even higher rate of early seizures that are often refractory to standard antiepileptic drugs. In a cohort of 80 infants with ISs, Alrifai et al. [126] identified an inborn or neurometabolic disorder in 12.5% of cases, showing that metabolic etiologies are not rare when actively searched for. The identified disorders included Leigh-like disease, ethylmalonic aciduria, non-ketotic hyperglycinemia due to *GCSH* deficiency, hyperinsulinemic hypoglycemia (*HHF17*), short-chain acyl-CoA dehydrogenase deficiency due to *ACADS* mutations, molybdenum cofactor deficiency caused by *GPHN*, primary carnitine deficiency due to *SLC22A5*, and hypoglycemia secondary to hypopituitarism (CPHD15). Other metabolic conditions in which ISs have been described comprise glycine encephalopathy caused by *GLDC* or *GCST* variants, DEND syndrome due to *KCNJ11* mutations, methylmalonic acidemia (*MUT*), maple syrup urine disease (*BCKDHA*, *BCKDHB*, *DBT*, *DLD*), and propionic acidemia (*PCCA*, *PCCB*). Neurodegenerative metabolic diseases such as Krabbe disease caused by *GALC* mutations and Menkes disease due to *ATP7A* defects may also present with ISs as part of their epileptic phenotype [127,128,129]. Rarer associations include cerebrotendinous xanthomatosis due to *CYP27A1* mutations [130], glucose transporter type 1 deficiency from *SLC2A1* exon 9 mutations [131], and congenital disorders of glycosylation related to *ALG1*, *ALG6*, or *ALG11* [132]. Pyridoxine-dependent epilepsy represents a special situation, since variants in *ALDH7A1* lead to accumulation of toxic intermediates and to seizures of multiple types, including ISs, which can respond dramatically to high-dose pyridoxine; however, atypical and late-onset forms with metabolic decompensation and EEG abnormalities have been reported, which makes the diagnosis more difficult [133,134]. These data justify a systematic metabolic work-up in infants with spasms, particularly when standard imaging is unrevealing.

Neurocutaneous disorders or phacomatoses provide an important link between genetic pathways regulating cell growth and the occurrence of ISs. These disorders typically present with a combination of congenital cutaneous lesions, ocular involvement, central or peripheral nervous system structural anomalies, and multiorgan features affecting the heart, kidney, or lung [135,136,137]. TSC represents the prototypical phacomatosis associated with ISs [138,139]. Pathogenic variants in *TSC1* or *TSC2* disrupt the hamartin–tuberin complex and cause constitutive activation of the mTOR pathway, which in turn impairs cortical lamination and interneuron development. This process results in cortical tubers, white matter radial bands, subependymal nodules, and sometimes subependymal giant cell astrocytoma, which provide multiple epileptogenic foci [112,140]. In a cohort of 81 children with TSC, 91% experienced seizures and 32% had a history of ISs, confirming that spasms are a frequent early seizure type in this condition [141]. Surgical resection of a dominant cortical tuber has been shown to reduce or abolish seizures, including spasms, in selected drug-resistant patients [142,143]. mTOR inhibitors, administered as monotherapy or in combination, have been proposed as disease-modifying agents for TSC-related epilepsy and ISs, although their long-term use is limited by adverse effects and by the observation that the antiepileptic response may wane over time [144,145,146].

Other phacomatoses can rarely present with ISs. In NF1, caused by loss of function of *NF1* and reduced activity of neurofibromin in the RAS–MEK–MAPK–ERK signaling cascade, epilepsy occurs in 5–6% of patients, although ISs account for only a small fraction and the overall prognosis of spasms in NF1 tends to be better than in structural or metabolic causes [135,137]. Sturge–Weber syndrome, which is caused by somatic activating variants in *GNAQ*, features capillary malformations of the face, leptomeningeal angiomatosis, and choroidal vascular involvement. Epilepsy develops in up to 77% of patients with unilateral and more than 90% with bilateral brain involvement, most often with focal seizures but occasionally with ISs when the cortical involvement is extensive and early [137]. These vascular neurocutaneous disorders illustrate that chronic cortical irritation, impaired venous drainage, and secondary cortical atrophy can also converge on the typical electroclinical pattern of ISs.

Overall, this body of evidence confirms that infantile spasms are not restricted to a single etiologic pathway but instead arise from diverse structural, chromosomal, metabolic, and neurocutaneous disorders that all impair early brain development. Identification of the precise cause is essential, since some of these conditions, particularly metabolic epilepsies and TSC, have targeted or disease-modifying treatments that may improve seizure control and developmental outcome when instituted promptly.

## 4. Pathogenesis and Molecular Mechanisms

### 4.1. Network-Level Pathophysiology: Disrupted Cortical–Subcortical Circuits

The pathophysiology of ISs is considered multifactorial and remains only partially understood, as diverse etiological factors can lead to the same electroclinical presentation (Table 2). Structural anomalies, genetic channelopathies, and acquired destructive lesions all appear to converge on a vulnerable stage of cortical and subcortical network development, producing the characteristic pattern of clustered spasms and hypsarrhythmia [66,147,148]. Structural anomalies such as lissencephaly, polymicrogyria, cortical tubers, and diffuse destructive conditions including hydranencephaly, together with genetically inherited channelopathies, have been identified as primary contributors to ISs. This suggests that the crucial factor is not the specific lesion type but its impact on early neuronal organization and on the maturation of cortical-subcortical circuits [66,147,148]. A widely accepted theoretical framework posits that early disruptions in neuronal and interneuronal connectivity lead to aberrant interactions between the neocortex, thalamus, basal ganglia, and brainstem. These network disturbances ultimately give rise to the hallmark symptoms of clustered spasms and hypsarrhythmia, even when MRI reveals only focal or unilateral abnormalities [148]. The clinical discrepancy between a localized injury and a generalized EEG pattern can be explained if a focal initiator recruits deep nuclei and thalamocortical pathways that propagate epileptic activity across both hemispheres, producing bilaterally symmetric spasms accompanied by widespread high-amplitude slow waves and spikes [149,150].

The association of ISs with cognitive impairment and autistic features can also be interpreted within this network framework. For example, deletions of *SCN2A* and *SCN3A* identified in a child with autism spectrum disorder and ISs reinforce the concept that shared genetic vulnerabilities may drive both abnormal cortical excitability and atypical cognitive or social development [151]. Similar observations across independent cohorts indicate that epileptic and neurobehavioral phenotypes may arise as parallel manifestations of an underlying developmental channelopathy or synaptopathy rather than as distinct complications [66,147]. This broader view places ISs within a spectrum of disorders characterized by impaired excitation–inhibition balance and disrupted large-scale neural integration.

ISs arise from dysfunction of distributed cortical–subcortical circuits rather than a single epileptogenic focus, and multimodal evidence consistently points to early involvement of deep structures that shape cortical excitability. Across human EEG-fMRI, resting-state fMRI, and peri-ictal EEG studies, thalamocortical pathways, cortico-striatal-thalamic loops, brainstem arousal systems, hippocampal–limbic structures, and large-scale interhemispheric networks repeatedly appear as nodes perturbed during hypsarrhythmia and spasm generation [192,193,194]. Notably, simultaneous EEG-fMRI demonstrates that BOLD activation begins in the brainstem and thalami before spreading to cortex [192], while GABAergic perturbations in a *GABRB3* knock-in mouse model prolong thalamocortical oscillatory firing and reduce cortical inhibition [194]. Lesion-network mapping in tuberous sclerosis further reveals that heterogeneous cortical lesions converge functionally on the bilateral globi pallidi, suggesting a shared basal-ganglia vulnerability irrespective of tuber location [192]. Complementing these deep-structure findings, resting-state graph-theory analyses show decreased clustering, reduced small-worldness, and increased global efficiency, reflecting a shift toward more randomized cortical topology and impaired hemispheric integration [193]. Collectively, these multimodal data support a model in which early deep-structure dysregulation propagates to a cortex rendered vulnerable by reduced inhibition and immature connectivity.

Mechanistically, converging data from genetics, developmental neurobiology, lesion studies, and rodent models indicate that impaired inhibition, altered thalamocortical dynamics, and dysfunctional network hubs together shape the electroclinical phenotype of ISs. In APC conditional-knockout mice, reduced numbers of parvalbumin-positive interneurons and abnormal interneuron maturation demonstrate a significant excitation–inhibition imbalance during the developmental window in which spasms emerge. These deficits parallel human EEG microphysiology, which shows pre-ictal cortical pauses resembling down-states followed by intense up-states that can trigger spasms and produce the characteristic electrodecrement. Furthermore, the temporal sequence of deep-structure activation, including the brainstem, thalamus, hippocampus, and basal ganglia, during hypsarrhythmia helps explain the diffuse cortical desynchronization and chaotic interictal EEG pattern observed in ISs [195]. Graph-theory markers of network randomization correlate with seizure frequency and may account for the cognitive and developmental regression commonly associated with ISs [193]. Peri-ictal analyses of fast-ripple activity on EEG reveal increased global efficiency and dynamic shifts in hub organization during spasms, reflecting rapid reconfiguration of cortical networks across ictal and interictal states [196,197,198,199].

Despite substantial progress, several gaps limit the translation of these network insights into targeted therapies. Human imaging studies remain predominantly correlative, and interventional or longitudinal multimodal designs are required to determine whether modifying identified hubs such as thalamic or pallidal circuits meaningfully alters clinical trajectories [192]. The absence of tract-level diffusion MRI and MEG studies constrains precise mapping of structural pathways and source localization of fast oscillations. Etiological heterogeneity further complicates mechanistic interpretation, highlighting the need for larger, stratified cohorts to differentiate network signatures associated with genetic, structural, and metabolic causes [192,193]. In parallel, mechanistic findings from animal models, including interneuron deficits and GABA-A receptor dysfunction, have not yet been translated into human trials [192,194]. Therefore, priority next steps include prospective longitudinal EEG-fMRI with standardized event modeling, integration of DTI-based tractography and MEG source localization, and early-phase interventional trials such as thalamic or pallidal neuromodulation or interneuron-targeted pharmacotherapies combined with network-level readouts to test causality and refine circuit-directed strategies for ISs.

### 4.2. Neurotrophin Dysregulation (BDNF, NGF, GDNF): Dual Role in Injury and Epileptogenesis

Neurotrophic signaling is increasingly recognized as an important contributor to the age-restricted vulnerability characteristic of ISs. Neurotrophins such as brain-derived neurotrophic factor (BDNF), nerve growth factor (NGF), glial cell line-derived neurotrophic factor (GDNF) and insulin-like growth factors (IGF)-1 and -2 modulate survival play essential roles in neuronal survival, differentiation, interneuron maturation, and early circuit formation, while also participating in immune–neuroendocrine interactions [152,153,154,155]. This developmental framework aligns with observations that ISs emerge during a critical maturational window in which disturbances in neurotrophin availability may disproportionately affect excitatory–inhibitory balance and network stability [200,201]. Clinical studies in neonates with hypoxic–ischemic injury—one of the most frequent antecedents of symptomatic ISs—report increased CSF BDNF and reduced CSF NGF, a pattern interpreted as a compensatory attempt to protect neurons from excitotoxic injury and as a marker of selective impairment of NGF-secreting systems [156,157,158,159]. However, although neurotrophins are conceptually linked to IESS pathogenesis, the supplied literature does not include primary mechanistic studies detailing canonical receptor–signaling cascades (e.g., BDNF/TrkB, NGF/TrkA, GDNF/GFRα-RET), regional developmental expression, or pathway-specific contributions to spasms. Thus, receptor-level mechanistic inferences remain insufficiently supported by available evidence.

Clinical and preclinical data nonetheless suggest that neurotrophin dysregulation accompanies ISs in several etiologies. CSF studies demonstrate that β-NGF levels vary markedly across etiologic groups. Infants with ISs due to tuberous sclerosis or postinfectious injury exhibit exceptionally high CSF NGF concentrations, far exceeding values observed in age-matched controls or cryptogenic cases, whereas infants with ISs of structural or hypoxic origin often show reduced NGF levels and poorer responsiveness to ACTH therapy [160,161,162,163,164,202,203]. This bidirectional profile supports a dual-role model in which either NGF deficiency (reflecting neuronal loss or impaired trophic support) or NGF excess (potentially driven by inflammation or mTOR pathway activation) can disrupt synaptic maturation and contribute to epileptogenesis. Elevated NGF in tuberous sclerosis and postinfectious ISs has led to the hypothesis that excessive NGF may actively promote hyperexcitability and could represent a future therapeutic target in these subtypes [165,166]. More broadly, reviews and animal-model syntheses propose that neurotrophins may modulate the transition from acute injury to chronic epileptogenesis by influencing synaptic plasticity, circuit reorganization, and inhibitory maturation, although direct, region-specific, temporally resolved neurotrophin measurements in validated IESS models are not provided in the supplied corpus [164,200].

The therapeutic implications of these findings remain preliminary. Conceptually, modulating neurotrophic signaling during the infantile critical window could either enhance recovery or prevent maladaptive remodeling, yet no clinical trials directly targeting neurotrophin pathways in ISs are reported in the available literature [164,200]. Existing treatment evidence focuses instead on standard therapies such as ACTH, which does not selectively influence neurotrophin pathways but remains effective in seizure cessation for many infants [203]. Because neurotrophin signaling is highly time-dependent, determining whether and when to enhance versus inhibit specific pathways is a key translational challenge that the current evidence base cannot resolve. Consequently, research priorities include longitudinal and regionally resolved measurement of BDNF, NGF, GDNF, and related molecules in animal models and human samples; experimental manipulation of TrkB, TrkA, and GFRα-RET pathways during defined developmental windows to test causality; and the design of larger etiologically stratified clinical cohorts to evaluate neurotrophins as biomarkers and to guide ethically and mechanistically informed neurotrophin-targeted therapeutic trials.

### 4.3. IGF-1 Deficiency and Impaired Steroid-Driven Trophic Signaling

IGF-1 is a key trophic mediator in early brain development, linking growth hormone signaling, mTOR activity, synaptogenesis, interneuron maturation, and resistance to cellular stress [170,171,172,173]. It supports neuronal survival, promotes synaptogenesis and myelination, and reduces microglial and astrocytic inflammation during the critical developmental window in which ISs emerge [202,204]. Although the supplied literature does not detail IGF-1′s primary structure or transcriptional regulation, consistent evidence demonstrates that IGF-1 receptor activation is essential for establishing inhibitory connectivity in neonatal cortex, a process central to preventing the excitatory–inhibitory imbalance characteristic of IESS [204]. Importantly, IGF-1 bioavailability differs from circulating IGF-1 levels: glucocorticoid exposure can increase serum IGF-1 while simultaneously reducing tissue-bioactive IGF-1 through IGFBP processing and post-receptor resistance mechanisms [201]. These compartment-specific effects underscore why trophic signaling may be impaired even when serum IGF-1 appears normal.

Clinical and experimental findings converge to show that IGF-1 deficiency contributes to the pathophysiology of ISs, particularly in spasms secondary to prenatal, perinatal, or early postnatal injury. Infants with ISs of unknown etiology often have CSF IGF-1 values comparable to controls and respond well to ACTH, whereas children with spasms following early structural insults show markedly reduced CSF IGF-1, poor hormonal response, and later cognitive decline [172,173]. Similar developmental vulnerabilities are seen in premature infants with low perinatal IGF-1, who are at elevated risk of neurodevelopmental delay [174,175]. Mechanistic support for these clinical patterns comes from the TTX neocortical lesion model, in which cortical IGF-1 expression is significantly reduced, accompanied by loss of GABAergic presynaptic terminals and impaired inhibitory connectivity; conditional reduction in IGF-1R reproduces these deficits, confirming a causal role for IGF-1 in interneuron maturation [204]. Human neocortical tissue resected after perinatal stroke shows the same reductions in IGF-1 and inhibitory markers, with regionally heterogeneous patterns, including reduced interneuronal IGF-1 but increased astrocytic IGF-1, suggesting both focal injury and downstream network-level deficits [204]. Notably, serum IGF-1, IGFBP-3, and their ratio correlate with short-term ACTH response and EEG characteristics in IESS, highlighting their potential as biomarkers for treatment responsiveness [202].

Therapeutically, the restoration of IGF-1 signaling shows robust disease-modifying potential in experimental models. Treatment with the IGF-1–derived tripeptide (1–3) IGF-1 rescues interneuron connectivity, normalizes inhibitory synapses, abolishes spasms, and reverses hypsarrhythmia-like EEG abnormalities in most treated animals [204]. In addition, this peptide reduced vigabatrin-related retinal toxicity in the CURE Infantile Spasms Consortium trial, further supporting translational feasibility [175]. The interaction between IGF-1 and hormonal therapies provides further mechanistic insight: ACTH acts not only by stimulating adrenal glucocorticoids but also through central melanocortin receptors such as MC2R, which modulate seizure susceptibility and influence ACTH efficacy [205,206]. Glucocorticoids can both enhance serum IGF-1 and impair tissue IGF-1 signaling, while non-neural studies show that glucocorticoids and IGF-1 can synergistically engage the PI3K/Akt pathway, potentially explaining ACTH’s superior clinical performance in some cohorts [207]. Indeed, registry data and trial syntheses show that ACTH, prednisolone, and vigabatrin each improve spasm cessation, but ACTH often yields greater electroclinical improvement and longer relapse-free intervals [207,208,209]. Together, these findings position IGF-1 deficiency as a mechanistic driver of interneuron and network dysfunction in ISs and support a therapeutic framework in which hormonal therapy, trophic signaling, and IGF-1-based interventions converge to restore developmental homeostasis.

### 4.4. GABAergic Immaturity and Neurosteroid Deficiency

Alterations in inhibitory neurotransmission constitute a major pathogenic pathway in ISs, reflecting the unique developmental trajectory of GABAergic signaling in early life. In the neonatal brain, GABA is initially depolarizing because intracellular chloride concentrations remain high, and only with maturation-driven upregulation of the *KCC2* chloride exporter does GABA_A receptor activation become hyperpolarizing and inhibitory [210]. Any process that delays or disrupts this developmental GABA_A “switch,” including impaired interneuron maturation, reduced *KCC2* expression, or receptor-level dysfunction, can sustain a state of network hyperexcitability during the age range when ISs appear [207]. The pathogenic potential of receptor abnormalities is underscored by a *GABRB3* beta3-subunit mutation that produces an IS-like phenotype in mice [211]. These mechanisms are consistent with clinical observations showing decreased GABA_A receptor expression and altered neurosteroid sensitivity in resected IS cortex [178,179,180], and with the therapeutic efficacy of vigabatrin, a GABA transaminase inhibitor that enhances synaptic GABA availability [212].

Human tissue studies and diverse animal models converge on a picture of interneuron immaturity, impaired inhibitory synaptic function, and chloride-handling deficits. Resected neocortex from infants with epileptic spasms demonstrates reduced *GAD*, parvalbumin, and synaptotagmin-2 expression, mirroring interneuron connectivity deficits in the TTX injury model [213]. Several genetic and acquired models provide etiologically specific support [214]. *APC* cKO mice display reduced PV-positive interneurons with altered inhibitory function at P9 (the peak spasm period) [215]. The *Arx*(GCG)10 + 7 model shows interneuron migratory defects and early postnatal apoptosis that can be rescued with neonatal estradiol or ACTH. The multiple-hit model indicates selective PV-interneuron loss, paralleling partial vigabatrin responsiveness in structural IS [207]. Environmental stressors can also influence GABAergic vulnerability, since prenatal stress increases susceptibility to NMDA-induced spasms and decreases *KCC2* and *GAD67* expression in offspring [208]. Although *NKCC1* likely contributes to early chloride homeostasis, the supplied corpus does not provide direct *NKCC1* measurements in IS, and the role of this transporter must therefore be regarded as insufficient evidence.

Neurosteroid biology provides an integrated mechanistic bridge between hormonal therapies and GABAergic maturation in ISs. Progesterone-derived metabolites such as 3-alpha-ol-20-one and endogenous steroids including THDOC act as potent positive allosteric modulators of GABA_A receptors, increasing both phasic and tonic inhibition while also reducing glutamatergic excitation [183,184,185]. These effects offer a compelling explanation for the rapid suppression of spasms following ACTH, which increases endogenous neurosteroid synthesis through adrenal and central melanocortin pathways [178,179,180,181,182]. ACTH nonresponders exhibit a low DHEA to androstenedione ratio, suggesting that impaired neurosteroidogenesis may reduce the efficacy of hormonal therapy [186]. Although direct measurements of neurosteroids or steroidogenic enzymes in human IS tissue are not provided in the available literature, indirect experimental support arises from the *ARX* model, where estradiol and ACTH restore interneuron density and suppress spasms. Broader neurosteroid studies further highlight the importance of CNS synthesis and of key enzymes such as 5-alpha-reductase and 3-alpha-hydroxysteroid dehydrogenase in regulating GABA_A receptor modulation [215,216].

Therapeutic implications follow from these converging pathways. Vigabatrin effectively increases brain GABA levels and is particularly beneficial in TSC-related IS, including presymptomatic seizure-prevention contexts [217]. However, models of structural-etiology IS show incomplete responsiveness, likely due to irreversible interneuron loss or transporter dysfunction. Hormonal therapies act through synergistic mechanisms related to anti-inflammatory effects, neurosteroidogenesis, and enhancement of GABA_A-mediated inhibition, and therefore complement GABAergic and chloride-stabilizing strategies [211]. Synthetic neurosteroids such as ganaxolone have been proposed for refractory ISs [188,189], although IS-specific clinical trial results are not included in the supplied corpus. Similarly, *NKCC1* inhibitors such as bumetanide lack IS-focused clinical evidence. Biomarker development remains an unmet need. CSF GABA levels are lower in symptomatic ISs than in idiopathic cases or controls, reflecting reduced inhibitory tone [187], yet validated biomarkers predicting response to vigabatrin, steroid therapy, or neurosteroid-targeted treatment are not available. Priority research directions include longitudinal studies of GABAergic and neurosteroid biomarkers, etiology-stratified translational models, and combination therapies that target chloride homeostasis, GABAergic stabilization, and trophic support [216].

### 4.5. Immune Activation, Inflammation, and mTOR Pathway in IS Epileptogenesis

The interaction of immune signaling with neurotrophic imbalance and inhibitory network immaturity provides a coherent mechanistic framework for IS epileptogenesis. In TSC, epileptogenic tubers contain activated microglia and reactive astrocytes that release cytokines and disturb local trophic support. Cerebrospinal fluid studies report elevated NGF and reduced IGF-1 in affected infants, creating a proconvulsant environment during early development. These abnormalities occur in parallel with dysregulated mTOR activity, GABAergic dysfunction, and impaired network maturation, all of which increase susceptibility to ISs [218].

**Figure 2:**
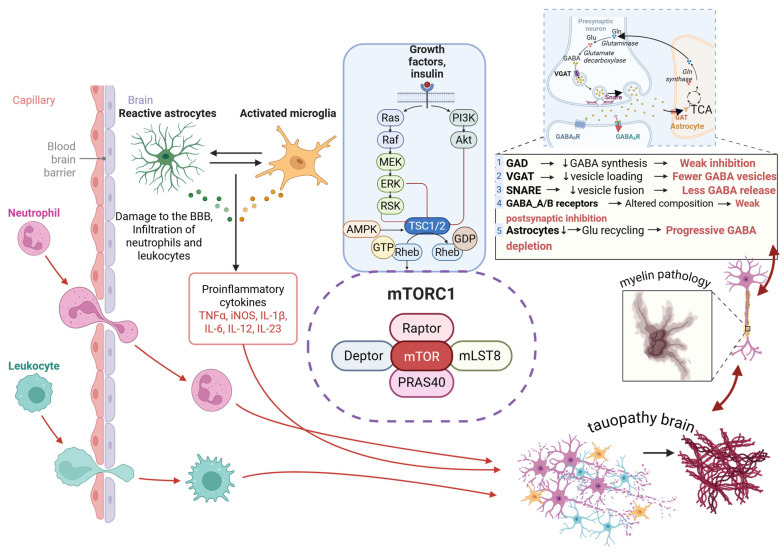
Immune activation, neuroinflammation, mTOR pathway dysregulation, and GABAergic impairment in IS epileptogenesis. Peripheral immune cells cross a compromised blood–brain barrier and interact with reactive astrocytes and activated microglia, leading to increased production of proinflammatory cytokines (TNFα, iNOS, IL-1β, IL-6, IL-12, IL-23). These cytokines amplify glial activation, oxidative stress, and alterations in ionic and neurotransmitter homeostasis. Upstream inflammatory signals engage the TSC1/2 complex and enhance mTORC1 activation, which disrupts interneuron development, synaptic maturation, and the balance of excitation and inhibition. Parallel defects in GABAergic transmission include reduced GAD activity, impaired vesicle loading via VGAT, decreased SNARE-dependent vesicle fusion, altered GABAA/GABAB receptor composition, and disrupted astrocytic glutamine–glutamate cycling. Together, these interacting pathways promote circuit instability, lower seizure thresholds, and facilitate the emergence of ISs [162,163,164,165,218]. Arrows indicate the direction of signaling and functional interactions between immune, glial, and neuronal pathways, highlighting pathogenic processes contributing to network dysfunction.

The interplay of neurotrophic imbalance, inhibitory dysfunction, immune activation, and mTOR pathway engagement creates a coherent framework for understanding IS epileptogenesis. In TSC, epileptogenic tubers contain activated microglia and reactive astrocytes that release cytokines, elevate CSF NGF, and reduce IGF-1, producing a proconvulsant and proinflammatory environment during early infancy [162,163,164,165]. A similar pattern appears in postinfectious IS, where systemic inflammation converges with immature GABAergic networks and enhances mTOR signaling. These inflammatory processes disrupt blood–brain barrier integrity, promote leukocyte entry, stimulate glial activation, alter glutamate and potassium buffering, and interfere with GABA synthesis, vesicle loading, and receptor trafficking. At the same time, mTORC1 hyperactivation affects interneuron development, synaptic maturation, and the balance between excitation and inhibition. Together, these events lower seizure thresholds and support the emergence of IS in vulnerable infants (Figure 2).

Growing evidence supports the presence of a proinflammatory neural environment during the early stages of IS. Human neuropathology and several animal models show early activation of microglia and astrocytes, which release cytokines and chemokines that influence neuronal excitability and synaptic maturation. Increased signaling through interleukin 1 receptor pathways and toll like receptors has been documented in developmental epileptic encephalopathies, including disorders marked by mTOR dysregulation [218]. Microglial proliferation and polarization toward inflammatory states increase extracellular glutamate, reduce debris clearance, and impair pruning mechanisms needed for healthy circuit development. Astrocyte dysfunction contributes to reduced glutamate and potassium buffering and abnormal GABA uptake, which destabilize inhibition. These mechanisms parallel findings in resected IS tissue where inflammatory gene expression is present even at early developmental stages. Together, these observations support the concept that neuroinflammation directly shapes the formation of epileptogenic networks in infancy.

Immune mediated contributions extend beyond resident glial cells. Blood–brain barrier dysfunction allows peripheral immune cells to enter the developing brain, where they release cytokines and free radicals. Studies in mTOR related disorders show albumin extravasation, endothelial activation, and metalloproteinase driven remodeling of the extracellular matrix [218]. These processes weaken barrier integrity and facilitate leukocyte trafficking. Perivascular astrocytes exposed to cytokines release mediators that increase oxidative stress and disturb ionic gradients. These events promote maladaptive synaptic remodeling and impair early functional connectivity, an effect that also appears in mosaic mTOR pathway disorders such as epidermal nevus syndromes, which frequently present with early-onset epilepsy and ISs [219]. Transcriptomic studies in infant brain tissue show increased expression of inflammatory transcription factors such as SPI1, indicating early microglial priming. In IS, the convergence of vascular inflammation, oxidative injury, and cytokine signaling contributes to widespread network disorganization and the abrupt emergence of hypsarrhythmia.

The mTOR pathway integrates immune activation, metabolic stress, and neurodevelopmental cues. Its dysregulation has emerged as a central driver of inflammation related epileptogenesis in IS. Hyperactivation of mTORC1 alters cell growth, interneuron development, and the balance of excitation and inhibition. Cytokines such as IL-1β and TNFα enhance these effects through upstream activation of PI3K–Akt signaling, suppression of autophagy, and promotion of oxidative stress. TSC provides a clear example of this mechanism. Prenatal mTOR overactivation in TSC leads to cortical malformations, persistent glial activation, disrupted myelination, and abnormal connectivity patterns that appear before seizure onset [218,220]. These abnormalities parallel defects in GABAergic maturation, including reduced GAD activity, altered vesicle loading, and impaired receptor trafficking. Similar mechanisms operate in neurocutaneous syndromes involving mosaic PI3K–Akt–mTOR mutations, where somatic pathway activation produces hemimegalencephaly and early-onset epileptic spasms [219]. Together, these data support a model in which immune activation and mTOR pathway overactivity act synergistically to impair neuronal circuit maturation, lower seizure thresholds, and drive the developmental epileptic encephalopathy that defines WS.

## 5. Therapeutic Approaches

The early detection and swift implementation of treatment remain the most pivotal factors influencing a positive prognosis in ISs. A plethora of clinical and population-based investigations have demonstrated that a reduced interval between the manifestation of spasms and the initiation of therapy significantly enhances seizure cessation rates, the resolution of hypsarrhythmia, and long-term neurodevelopmental outcomes [190,191]. Delays extending beyond four weeks from the initial presentation are consistently associated with diminished treatment responsiveness and an elevated risk of subsequent refractory epilepsy and cognitive deficits. The fundamental therapeutic principle is the early disruption of epileptogenic network activity prior to irreversible cortical remodeling. Once the electroclinical diagnosis is confirmed, therapeutic intervention should commence immediately, even before the complete clarification of etiology. The initial diagnostic approach frequently involves a trial of pyridoxine (vitamin B6), administered intravenously at a dosage of 100–150 mg over 5–10 min under electrocardiographic surveillance to exclude pyridoxine-dependent epilepsy, a treatable metabolic disorder linked to mutations in the *ALDH7A1*, *PLPBP*, or *PNPO* genes. In these instances, a notable clinical and electroencephalographic enhancement typically occurs within hours of administration [221]. Should no improvement occur, the infusion should be halted to avert toxicity. Some authorities advocate for a subsequent trial of pyridoxal-5′-phosphate in cases of suspected *PNPO* deficiency, especially in neonates with metabolic irregularities.

Concurrent biochemical analysis for α-aminoadipic semialdehyde and pipecolic acid in plasma or urine may further substantiate the diagnosis of vitamin B6-dependent epilepsies. When these etiologies are ruled out, disease-modifying therapy employing ACTH, corticosteroids, or vigabatrin should be promptly instituted. Findings from the National Infantile Spasms Consortium indicate that the commencement of therapy within two weeks of the onset of spasms nearly doubles the probability of achieving complete electroclinical remission compared to delayed treatment, and early responders tend to exhibit superior adaptive and cognitive development by 12 to 24 months, irrespective of the underlying etiology. Consequently, the prevailing management paradigm underscores the urgency of rapid recognition, immediate initiation of targeted therapy, and timely transition from diagnostic to disease-modifying interventions to avert permanent synaptic and neurodevelopmental compromise [220].

The foundation of pharmacological intervention in ISs continues to be the synergistic application of hormonal therapies alongside GABAergic modulation, which collectively seek to mitigate epileptiform activity and reestablish homeostatic equilibrium within the maturing brain. Within the category of hormonal agents, ACTH, corticosteroids (notably prednisolone), and vigabatrin form the primary therapeutic triad [190,191,221,222]. ACTH demonstrates pleiotropic effects on corticotropin receptors located in both the adrenal cortex and the central nervous system, resulting in enhanced glucocorticoid synthesis, inhibition of corticotropin-releasing hormone (CRH), which is a recognized proconvulsant peptide, and modulation of inflammatory cytokines associated with epileptogenesis. Despite the considerable variability in ACTH dosing protocols across different medical institutions, recent meta-analyses and multicenter investigations have indicated comparable efficacy between low-dose (20–30 IU/day) and high-dose (75–150 IU/day) treatment regimens, suggesting that lower dosages can yield equivalent electroclinical remission with a reduced incidence of metabolic and cardiovascular complications [223]. The standard therapeutic regimen typically encompasses a 2–4-week administration of intramuscular ACTH, followed by a gradual tapering phase, frequently transitioning to oral corticosteroids to avert relapse [223,224,225].

Prednisolone, which operates via glucocorticoid receptor-mediated transcriptional mechanisms, attenuates neuroinflammation and stabilizes hyperexcitable cortical networks. Several controlled trials, including the United Kingdom Infantile Spasms Study (UKISS) and subsequent randomized studies, have demonstrated that high-dose oral prednisolone (40–60 mg/day for 2 weeks) exhibits comparable efficacy to intramuscular ACTH in facilitating early cessation of spasms and normalization of EEG, thereby positioning it as a viable and cost-effective first-line alternative in settings with limited resources [226,227]. Additionally, the exploration of combination therapy utilizing hormonal agents alongside vigabatrin has been investigated as a synergistic strategy, revealing elevated short-term remission rates in specific cohorts, although the long-term developmental advantages remain inconsistent across various studies [228].

Adjunctive and non-hormonal therapies provide additional options for refractory or etiology-specific cases. Vigabatrin, an irreversible inhibitor of GABA transaminase, increases GABAergic inhibition and is particularly effective in ISs associated with tuberous sclerosis complex, achieving spasm cessation in up to 70 percent of cases. Its long-term use, however, carries a risk of retinal toxicity and reversible MRI signal changes in deep gray matter structures, which necessitates careful ophthalmologic and imaging surveillance [229]. Ketogenic dietary therapies represent another nonpharmacological option for drug-resistant ISs. The classical ketogenic diet, high in fat and low in carbohydrates, promotes ketone body production that stabilizes neuronal networks through enhanced mitochondrial function, increased GABA synthesis, and reduced oxidative stress. Clinical studies report complete or substantial seizure reduction in 40 to 60 percent of treated patients [227]. Modified dietary approaches, including medium-chain triglyceride and modified Atkins diets, offer improved tolerability and similar effectiveness [230]. These dietary therapies show particular benefit in metabolic etiologies such as pyruvate dehydrogenase deficiency and *GLUT1* deficiency syndrome, where they function as disease-specific metabolic treatments. Together, these strategies demonstrate the growing importance of aligning therapy with underlying biochemical and metabolic mechanisms, which supports a precision-oriented approach.

When spasms persist despite first-line therapy, an early transition to a second-line agent is recommended. The choice of subsequent therapies depends on the suspected underlying mechanism. Sodium valproate, topiramate, levetiracetam, clobazam, and zonisamide have been used as monotherapies or adjuncts, achieving partial or complete remission in 20 to 40 percent of refractory cases [231,232,233]. Valproate remains widely accessible but requires monitoring for hepatotoxicity and mitochondrial dysfunction, particularly in children with *POLG* mutations [234]. Multicenter data show that roughly one-third of nonresponders achieve remission with a mechanistically distinct second-line therapy, highlighting the critical role of mechanism-based treatment sequencing [235].

Precision-oriented therapeutic strategies are becoming increasingly relevant in ISs as genetic and molecular findings begin to inform treatment selection. Everolimus and other mTOR inhibitors demonstrate significant benefit in patients with *TSC1* or *TSC2* mutations by reducing mTOR overactivation and improving seizure control [208]. Memantine has been explored in cases with pathogenic variants in *GRIN2A* or *GRIN2B*, where excessive NMDA receptor activity contributes to epileptogenesis, while retigabine provides a mechanistic match for disorders involving *KCNQ2* or *KCNQ3* channel dysfunction [235,236]. Neurosteroid analogs such as ganaxolone, which enhance GABAA receptor signaling, and IGF-1 derived peptides that support synaptic maturation, exemplify therapies guided by specific neurochemical deficits. Emerging approaches include antisense oligonucleotides and CRISPR-based strategies that aim to correct pathogenic variants in *CDKL5* and *ARX*, although these remain in preclinical stages. These innovations mark a transition from empirical treatment toward biomarker-driven precision therapy, where genomic, transcriptomic, and metabolomic findings guide drug selection and predict treatment response.

Future priorities include establishing validated biomarkers that can stratify patients based on predicted therapy responsiveness, integrating molecular diagnostics into treatment algorithms, and designing clinical trials that evaluate etiology-specific interventions. Collectively, the expanding therapeutic landscape signals a shift away from generalized seizure suppression toward targeted modification of the underlying molecular pathology, offering the potential for improved developmental outcomes and durable remission.

## 6. Future Perspectives

Future directions in the management of ISs will hinge upon elucidating the reasons behind the differential responses of infants to ACTH or prednisolone, particularly in cases where treatment is initiated at an early stage. Pioneering studies comparing high-dose ACTH with prednisone demonstrated the efficacy of hormonal therapies; however, such efficacy is not universally applicable, suggesting that the influence of hormones on CRH, neurosteroids, and inflammatory mediators varies among individuals. This phenomenon necessitates a re-examination utilizing contemporary methodologies, ensuring that ACTH treatment is guided by biological predictors of response rather than applied empirically. It is imperative to prioritize prospective investigations that correlate early endocrine and CSF profiles with both seizure cessation and normalization of EEG readings, employing the standardized diagnostic and therapeutic frameworks previously established by the AAN/CNS, and ILAE for ISs [43,48,52,55].

A secondary approach involves the comprehensive incorporation of expedited genetic testing into the initial management pathway for infants exhibiting ISs. Several of the most commonly implicated genes associated with ISs, including *STXBP1*, *KCNQ2*, *GRIN2A*, as well as the TSC-related genes *TSC1* and *TSC2*, are already classified as actionable or partially actionable. This indicates that identification of the specific molecular anomaly can guide therapeutic decisions regarding hormonal treatments, vigabatrin, mTOR inhibitors, or early metabolic and dietary interventions [28,68,234]. Consequently, clinical pathways ought to be realigned to ensure that array-CGH and NGS are conducted concurrently with hormonal therapy rather than subsequent to multiple pharmacological failures. This concurrent approach will facilitate the earlier transition to disease-modifying treatments for TSC-associated ISs and to targeted receptor or channel therapies for glutamatergic or potassium channel-related epileptic encephalopathies.

A tertiary, closely associated consideration is the formulation of combination therapies that address multiple disrupted pathways simultaneously. Evidence from ICISS and other multicenter studies indicates that the addition of vigabatrin to hormonal treatment enhances the early electroclinical response in specific subpopulations, although not uniformly across all developmental metrics. This finding implies that the GABAergic deficiency, along with neuroinflammatory or CRH-driven factors, should be concurrently addressed rather than sequentially [48,51]. Subsequent clinical trials should evaluate rational combination therapies such as ACTH combined with vigabatrin, ACTH with a ketogenic diet, or hormonal therapy paired with IGF-1 analogs in conditions where imbalances in IGF-1 and neurotrophins are established, utilizing predefined biomarkers for candidate selection and toxicity monitoring.

Lastly, precision and restorative therapeutic strategies must be implemented during early life stages, rather than restricted to refractory cases. The promising outcomes associated with everolimus in TSC-related epilepsy demonstrate that mTOR inhibition can mitigate seizure frequency when the underlying causal mechanisms are identified; similar reasoning applies to NMDA receptor antagonism in *GRIN2A*-associated ISs and to KCNQ openers in *KCNQ2* epileptic encephalopathy [234,235,236]. The subsequent phase involves integrating these targeted pharmacological agents with longitudinal neurodevelopmental assessments, ensuring that seizure control is not the sole outcome measure. Multicenter registries, standardized outcome metrics, and early genetic stratification will be critical in demonstrating that pathogenetic, rather than merely symptomatic, interventions for ISs can enhance long-term cognitive, linguistic, and social adaptation outcomes.

## 7. Conclusions

ISs constitute a multifaceted, intricate epileptic encephalopathy wherein various genetic, structural, metabolic, and immunological factors converge upon a singular pathway of network dysregulation during a pivotal developmental phase. Despite the diverse origins contributing to its manifestation, the commonality lies in the premature disruption of the inhibitory and excitatory equilibrium within cortical-subcortical circuits, which is foundational to the electroclinical manifestation of hypsarrhythmia and the ensuing neurodevelopmental deterioration. Recent advancements in neuroimaging, molecular genetics, and neurochemical profiling have markedly enhanced the categorization of ISs, uncovering that genetic etiologies, including alterations in *STXBP1*, *KCNQ2*, *GRIN2A*, and *TSC1*/*TSC2*, are significantly more prevalent than previously acknowledged. The incorporation of high-throughput genomic diagnostics such as NGS and WES has facilitated the earlier identification of actionable genetic variants, thereby transforming the diagnostic framework from purely descriptive to mechanistic, thus bolstering the nascent paradigm of precision-targeted therapy.

Timely identification and prompt commencement of therapeutic interventions remain fundamental to achieving favorable outcomes. Hormonal therapies, including ACTH and prednisolone, alongside vigabatrin, persist as primary treatment modalities capable of disrupting epileptogenic activity and enhancing developmental trajectories when initiated within the initial weeks following onset. Supplementary strategies, incorporating ketogenic dietary therapy and neurosteroid-based interventions, offer additional pathways for seizure management, particularly in refractory cases or distinct etiological subgroups. Crucially, expedited transition from diagnostic assessment to disease-modifying treatment has been demonstrated to significantly enhance the probability of remission and to alleviate long-term cognitive ramifications, underscoring the imperative of therapeutic responsiveness as a vital factor influencing prognosis.

The therapeutic landscape surrounding ISs is progressively evolving towards targeted molecular and restorative strategies aimed at altering the fundamental disease biology, rather than solely focusing on seizure suppression. The successful implementation of mTOR inhibitors in TSC-associated ISs exemplifies how elucidating specific pathogenic pathways can yield efficacious, mechanism-oriented therapies. Similarly, novel pharmacological agents such as NMDA receptor antagonists for *GRIN2A*/*GRIN2B* mutations, KCNQ channel openers for *KCNQ2*-related epileptic encephalopathies, and IGF-1 analogues for trophic deficiency syndromes signify the forthcoming generation of personalized treatments. Preclinical investigations into antisense oligonucleotides and gene-editing technologies, including CRISPR-Cas9, present the potential to rectify causative mutations at their inception, marking a pivotal shift towards curative molecular medicine.

Ultimately, the management of ISs necessitates a multidisciplinary and progressive framework that integrates early clinical vigilance, thorough molecular diagnostics, and mechanistically informed interventions. The convergence of neurogenetic research, advanced neuroimaging, and neuroendocrine studies is poised to enhance our comprehension of how structural, synaptic, and biochemical anomalies yield the shared phenotype of ISs. By aligning precision medicine with early intervention strategies, the discipline is positioned to transform ISs from a devastating early-life encephalopathy into a manageable condition characterized by improved seizure control, preserved cognitive potential, and enhanced quality of life for affected children and their families.

## Figures and Tables

**Table 1:** Most frequently reported genes and loci associated with WS.

No.	Gene	Cytogenetic Location	Inheritance/Pattern (Typical)	Comment/Note	Ref.
1	*ARX*	Xp21.3	X-linked, often de novo	Classic ISs gene, migration and interneuron defect	[69]
2	*CDKL5*	Xp22.13	X-linked, often de novo	Early-onset epileptic spasms, severe DD	[70]
3	*PAFAH1B1*/*LIS1*	17p13.3	AD, de novo	Lissencephaly, classic structural-spasm link	[71]
4	*DCX*	Xq23	X-linked, de novo	Lissencephaly/subcortical band heterotopia	[72]
5	*TUBA1A*	12q13.12	AD, de novo	Tubulinopathy with cortical malformation	[73]
6	*STXBP1*	9q34.11	AD, de novo	Common single-gene cause of IESS	[74]
7	*KCNQ2*	20q13.33	AD, de novo	Severe neonatal epileptic encephalopathy	[75]
8	*MAGI2*	7q11.23	AD, CNV/deletion	Reported in ISs, 7q11.23 region	[76]
9	*GRIN2A*	16p13.2	AD, de novo/familial	Glutamatergic receptor, ISs and other DEE	[77]
10	*GRIN2B*	12p13.1	AD, de novo	Early-onset DEE with spasms	[78]
11	*FOXG1*	14q12	AD, de novo	Postnatal microcephaly, spasms reported	[79]
12	*NSD1*	5q35.3	AD, de novo	Sotos phenotype, seizures/spasms	[80]
13	*SPTAN1*	9q34.11	AD, de novo	Spasms and hypomyelination	[81]
14	*NEDD4*	15q21.3	AD, CNV	Potential risk factor in ISs	[82]
15	*CALN1*	7q11.22	AD, CNV/intronic deletion	Risk factor in ISs CNV studies	[83]
16	*WDR45*	Xp11.23	X-linked, de novo	Neurodegeneration with brain iron accumulation	[84]
17	*RARS2*	6p21.1	AR	Pontocerebellar hypoplasia, spasms	[85]
18	*UBA5*	3q22.1	AR	Early infantile epileptic encephalopathy	[86]
19	*IARS2*	1q41	AR	CAGSSS spectrum, spasms	[87]
20	*PHACTR1*	6p24.1	AD, de novo	Candidate gene in ISs cohort	[88]
21	*ATP2A2*	12q24.11	AD	Novel candidate gene in Chinese cohort	[89]
22	*CD99L2*	Xq28	X-linked	Candidate gene in IESS cohort	[90]
23	*CLCN6*	1p36.22	AD, de novo	Epileptic encephalopathy with spasms	[91]
24	*CYFIP1*	15q11.2	AD, CNV	Developmental delay and seizures	[92]
25	*CYFIP2*	5q33.3	AD, de novo	DEE with spasms	[93]
26	*GNB1*	1p36.33	AD, de novo	DEE with hypotonia and spasms	[94]
27	*GPT2*	16q21	AR	Metabolic DEE with spasms	[95]
28	*HUWE1*	Xp11.22	X-linked	Intellectual disability and epilepsy	[96]
29	*KMT2D*	12q13.12	AD	Kabuki spectrum, seizures in infancy	[97]
30	*MYO18A*	17q11.2	AD/AR	Candidate gene in Chinese cohort	[98]
31	*NOS3*	7q36.1	AD	Candidate variant, possible modifier	[99]
32	*RYR1*	19q13.2	AD/AR	Ca^2+^ signaling, candidate gene	[100]
33	*RYR2*	1q43	AD	Ca^2+^ release channel, candidate gene	[101]
34	*RYR3*	15q13.3–q14	AD	Candidate gene in 2020s cohorts	[102]
35	*TAF1*	Xq13.1	X-linked	DEE with early spasms	[103]
36	*TECTA*	11q23.3	AD	Candidate gene in WES screen	[104]
37	*PURA*	5q31.3	AD, de novo	PURA syndrome, seizures and spasms	[105]
38	*SCN2A*	2q24.3	AD, de novo	Common channel gene in IESS cohorts	[106]
39	*SCN1A*	2q24.3	AD, de novo/familial	ISs with Dravet-like features	[107]
40	*SCN8A*	12q13.13	AD, de novo	Early-onset DEE, spasms described	[108]
41	*WWOX*	16q23.1	AR	WWOX-related encephalopathy with spasms	[109]
42	*SLC35A2*	Xp11.23	Somatic/germline, X-linked	Mosaic ISs with focal dysplasia	[110]
43	*NF1*	17q11.2	AD	NF1 with early-onset epileptic spasms	[111]
44	*TSC2*/*TSC1*	16p13.3/9q34	AD, de novo/familial	Frequent syndromic cause of IESS	[112]
45	*TOP2B*	3p24.3	AD, de novo	Emerging gene, single recent report	[113]

*ARX*, aristaless related homeobox; *CDKL5*, cyclin dependent kinase like 5; *PAFAH1B1/LIS1*, platelet activating factor acetylhydrolase 1B subunit 1 (lissencephaly 1); *DCX*, doublecortin; *TUBA1A*, tubulin alpha-1A; *STXBP1*, syntaxin-binding protein 1; *KCNQ2*, potassium voltage-gated channel subfamily Q member 2; *MAGI2*, membrane associated guanylate kinase inverted 2; *GRIN2A*, glutamate ionotropic receptor NMDA type subunit 2A; *GRIN2B*, glutamate ionotropic receptor NMDA type subunit 2B; *FOXG1*, forkhead box G1; *NSD1*, nuclear receptor-binding SET domain protein 1; *SPTAN1*, spectrin alpha, non-erythrocytic 1; *NEDD4*, neural precursor cell-expressed developmentally down-regulated protein 4 E3 ubiquitin-protein ligase; *CALN1*, calneuron 1; *WDR45*, WD repeat domain phosphoinositide-interacting protein 45; *RARS2*, arginyl-tRNA synthetase 2 (mitochondrial); *UBA5*, ubiquitin-like modifier activating enzyme 5; *IARS2*, isoleucyl-tRNA synthetase 2 (mitochondrial); *PHACTR1*, phosphatase and actin regulator 1; *ATP2A2*, ATPase sarcoplasmic/endoplasmic reticulum Ca^2+^ transporting 2; *CD99L2*, CD99 molecule-like 2; *CLCN6*, chloride voltage-gated channel 6; *CYFIP1*, cytoplasmic FMR1-interacting protein 1; *CYFIP2*, cytoplasmic FMR1-interacting protein 2; *GNB1*, G protein beta subunit 1; *GPT2*, glutamate-pyruvate transaminase 2; *HUWE1*, HECT, UBA, WW domain-containing E3 ubiquitin-protein ligase 1; *KMT2D*, lysine methyltransferase 2D (Kabuki syndrome gene); *MYO18A*, myosin XVIII A; *NOS3*, nitric oxide synthase 3 (endothelial); *RYR1*, ryanodine receptor 1 (skeletal muscle); *RYR2*, ryanodine receptor 2 (cardiac); *RYR3*, ryanodine receptor 3 (neuronal); *TAF1*, TATA-box binding protein-associated factor 1; *TECTA*, tectorin alpha; *PURA*, purine-rich element-binding protein A; *SCN2A*, sodium voltage-gated channel alpha subunit 2; *SCN1A*, sodium voltage-gated channel alpha subunit 1; *SCN8A*, sodium voltage-gated channel alpha subunit 8; *WWOX*, WW domain-containing oxidoreductase; *SLC35A2*, solute carrier family 35 member A2; *NF1*, neurofibromin 1; *TSC2/TSC1*, tuberous sclerosis complex genes (hamartin and tuberin); *TOP2B*, DNA topoisomerase II beta; AD, autosomal dominant; AR, autosomal recessive; CNV, copy number variant; X-linked, mutation located on the X chromosome; DEE, developmental and epileptic encephalopathy; IESS, infantile epileptic spasms syndrome; DD, developmental delay; CAGSSS, cataract–ataxia–short stature–skeletal dysplasia–seizures syndrome; WES, whole-exome sequencing; WGS, whole-genome sequencing; CGH, comparative genomic hybridization.

**Table 2:** Pathogenetic and molecular mechanisms: neurotrophic, hormonal, and neurotransmitter interactions.

No.	Pathogenetic/ Molecular Factor	Key Abnormality/Proposed Mechanism	Clinical or Etiologic Context	Therapeutic Implication/Target	Ref.
1	Disruption of cortical–subcortical networks	Early distortion of neuronal and interneuronal connectivity causes abnormal interaction between cortex, thalamus, basal ganglia and brainstem. Focal lesion can generate generalized spasms and hypsarrhythmia	Lissencephaly, polymicrogyria, cortical tubers, hydranencephaly, focal cortical lesions with generalized EEG	Early lesion localization and resection in focal cases; rationale for treating even when MRI looks focal	[66,147,148,149,150,151]
2	Genetic background for excitability and neurobehavioral phenotype	Variants in *SCN2A*, *SCN3A* and other developmental genes predispose to both epileptic spasms and ASD or cognitive delay through a shared channelopathy/synaptopathy	ISs with autism spectrum features or early developmental delay even before spasms	Genetic testing to define predisposition; possible future precision therapy	[66,147]
3	Neurotrophin imbalance (NGF, BDNF, GDNF)	After hypoxic or inflammatory injury CSF BDNF increases while NGF may decrease. In TSC or postinfectious ISs NGF can be excessively high. Both deficiency and excess can disturb synaptic maturation	Hypoxic–ischemic encephalopathy, postinfectious ISs, TSC with epileptic tubers	NGF modulation as experimental target; explains variable ACTH response	[152,153,154,155,156,157,158,159,160,161,162,163,164]
4	Low NGF in destructive or severe structural ISs	Low CSF NGF correlates with poor ACTH response and more extensive neuronal loss, probably due to limited capacity for synaptic repair	ISs with known structural or hypoxic etiology and delayed development	Early hormonal therapy before severe loss; marker of poor prognosis	[160,161,162,163,164,165,166,167,168,169,170,171]
5	IGF-1 deficiency and impaired steroid driven trophic support	Early stress, perinatal brain damage or ischemia reduces CSF IGF-1 and ACTH. This prevents mTOR-mediated survival, synaptogenesis and anti-inflammatory effects and favors epileptogenesis	Symptomatic ISs after prenatal/perinatal insults; premature infants; ISs with cerebral atrophy	IGF-1 or IGF-1 tripeptide (1–3) as adjunct; ACTH, steroids, ketogenic diet partly act through IGF-1	[172,173,174,175,176,177]
6	Preserved IGF-1 in cryptogenic/idiopathic ISs	Normal CSF IGF-1 in infants with unknown etiology correlates with good ACTH response and better cognitive outcome	ISs with normal MRI and no early insult	Usual first line hormonal therapy is adequate	[172]
7	Experimental evidence of IGF-1 rescue	In TTX model of ISs loss of IGF-1 and astrogliosis mimicked human ISs. IGF-1 (1–3) restored inhibitory neurons, stopped spasms and normalized EEG	Experimental ISs, neonatal stroke, postsurgical epileptic spasms	IGF-1 analogues and trofinetide are promising; can reduce vigabatrin retinal toxicity	[171,173]
8	Delay or failure of GABAA developmental switch	GABA remains depolarizing in infancy if the switch is delayed. This maintains network hyperexcitability at the age when ISs occur	Early life epilepsies, symptomatic ISs, TSC, postinfectious ISs	Make GABA more effective: vigabatrin, ACTH (via neurosteroids), ketogenic diet	[178,179,180,181,182]
9	Neurosteroid deficit	Reduced production of endogenous steroids gives poor enhancement of GABAA receptors. Low DHEA/androstenedione ratio seen in non-responders to ACTH	ACTH poor responders, symptomatic ISs	Pharmacologic neurosteroids such as ganaxolone	[180,181,182,183,184,185,186,187,188]
10	Reduced CSF GABA in symptomatic ISs	Symptomatic etiologies show lower CSF GABA than idiopathic cases and controls, which confirms insufficient inhibitory tone	Structural/metabolic ISs with poor development	Supports use of GABAergic drugs and neurosteroid based strategies	[187,188,189,190,191]
11	Inflammatory and mTOR related epileptogenesis	In TSC and in postinfectious ISs inflammatory cells, cytokines and mTOR activation are found around epileptogenic lesions. This is paralleled by high NGF and low IGF-1 which favor seizures	TSC, postinfectious ISs, cortical tubers, mTORopathies	mTOR inhibitors, anti-inflammatory strategies, NGF modulation as adjuvant	[162,163,164,165]
12	Converging networks hypothesis	Different primary hits (genetic, structural, metabolic, inflammatory) act on the same immature network where neurotrophins, GABA, IGF-1 and HPA axis are interlinked. Any disruption gives the same hypsarrhythmic output	Explains why ISs arise from many different causes	Justifies use of ACTH, steroids, ketogenic diet, and possibly IGF-1 analogues although triggers differ	[66,147,178]

## Abbreviations

The following abbreviations are used in this manuscript:

ACADS	Acyl-CoA dehydrogenase, C-2 to C-3 short chain
ACTH	Adrenocorticotropic hormone
AD	Autosomal dominant
AAN	American Academy of Neurology
AASA	α-Aminoadipic semialdehyde
ALG13	Asparagine-linked glycosylation 13
ARX	Aristaless related homeobox
ATP2A2	ATPase sarcoplasmic/endoplasmic reticulum Ca^2+^ transporting 2
B3GALNT2	Beta-1,3-N-acetylgalactosaminyltransferase 2
CAGSSS	Cataract–ataxia–short stature–skeletal dysplasia–seizures syndrome
CDKL5	Cyclin-dependent kinase-like 5
CNV	Copy number variation
CNS	Central nervous system
DCX	Doublecortin
DEE	Developmental and epileptic encephalopathy
EEG	Electroencephalography
FOXG1	Forkhead box G1
GABA	Gamma-aminobutyric acid
GDNF	Glial cell line-derived neurotrophic factor
GRIN2A	Glutamate ionotropic receptor NMDA type subunit 2A
GRIN2B	Glutamate ionotropic receptor NMDA type subunit 2B
IESS	Infantile epileptic spasms syndrome
IGF-1	Insulin-like growth factor 1
ILAE	International League Against Epilepsy
ISs	Infantile spasms
KCNQ2	Potassium voltage-gated channel subfamily Q member 2
LIS1	Platelet activating factor acetylhydrolase 1B subunit 1 (lissencephaly 1)
MAGI2	Membrane associated guanylate kinase inverted 2
mTOR	Mechanistic target of rapamycin
NF1	Neurofibromin 1
NGF	Nerve growth factor
NSD1	Nuclear receptor-binding SET domain protein 1
PKU	Phenylketonuria
PNPO	Pyridox(am)ine 5′-phosphate oxidase
RARS2	Arginyl-tRNA synthetase 2 (mitochondrial)
RYR1	Ryanodine receptor 1 (skeletal muscle)
RYR2	Ryanodine receptor 2 (cardiac)
RYR3	Ryanodine receptor 3 (neuronal)
SCN1A	Sodium voltage-gated channel alpha subunit 1
SCN2A	Sodium voltage-gated channel alpha subunit 2
SCN8A	Sodium voltage-gated channel alpha subunit 8
STXBP1	Syntaxin-binding protein 1
TSC	Tuberous sclerosis complex
TSC1	Tuberous sclerosis complex 1 (hamartin)
TSC2	Tuberous sclerosis complex 2 (tuberin)
VGB	Vigabatrin
WDR45	WD repeat domain phosphoinositide-interacting protein 45
WES	Whole-exome sequencing
WGS	Whole-genome sequencing
WS	West syndrome
WWOX	WW domain-containing oxidoreductase
ZNHIT3	Zinc finger HIT-type containing 3
UKISS	United Kingdom Infantile Spasms Study
